# Radiation Increases Bioavailability of Lisinopril, a Mitigator of Radiation-Induced Toxicities

**DOI:** 10.3389/fphar.2021.646076

**Published:** 2021-04-27

**Authors:** Meetha Medhora, Preeya Phadnis, Jayashree Narayanan, Tracy Gasperetti, Jacek Zielonka, John E. Moulder, Brian L. Fish, Aniko Szabo

**Affiliations:** ^1^ Department of Radiation Oncology, Medical College of WI, Milwaukee, WI, United States; ^2^ Department of Medicine, Medical College of WI, Milwaukee, WI, United States; ^3^ Department of Physiology, Medical College of WI, Milwaukee, WI, United States; ^4^ Cardiovascular Center, Medical College of WI, Milwaukee, WI, United States; ^5^ Research Service, Department of Veterans Affairs, Zablocki VAMC, Milwaukee, WI, United States; ^6^ GlobalReach BI, San Francisco, CA, United States; ^7^ Department of Biophysics, Medical College of WI, Milwaukee, WI, United States; ^8^ Cancer Center Redox and Bioenergetics Shared Resource, Medical College of WI, Milwaukee, WI, United States; ^9^ Institute for Health and Equity, Division of Biostatistics, Medical College of WI, Milwaukee, WI, United States

**Keywords:** pharmacokinetics, renin-angiotensin system, delayed effects of radiation, pulmonary vasculature, mitigation

## Abstract

There are no FDA-approved drugs to mitigate the delayed effects of radiation exposure that may occur after a radiological attack or nuclear accident. To date, angiotensin-converting enzyme inhibitors are one of the most successful candidates for mitigation of hematopoietic, lung, kidney, and brain injuries in rodent models and may mitigate delayed radiation injuries after radiotherapy. Rat models of partial body irradiation sparing part of one hind leg (leg-out PBI) have been developed to simultaneously expose multiple organs to high doses of ionizing radiation and avoid lethal hematological toxicity to study the late effects of radiation. Exposures between 9 and 14 Gy damage the gut and bone marrow (acute radiation syndrome), followed by delayed injuries to the lung, heart, and kidney. The goal of the current study is to compare the pharmacokinetics (PK) of a lead angiotensin converting enzyme (ACE) inhibitor, lisinopril, in irradiated vs. nonirradiated rats, as a step toward licensure by the FDA.

**Methods:** Female WAG/RijCmcr rats were irradiated with 12.5–13 Gy leg-out PBI. At day 35 after irradiation, during a latent period for injury, irradiated and nonirradiated siblings received a single gavage (0.3 mg, 0.6 mg) or intravenous injection (0.06 mg) of lisinopril. Plasma, urine, lung, liver and kidney levels of lisinopril were measured at different times. PK modeling (R package) was performed to track distribution of lisinopril in different compartments.

**Results:** A two-compartment (central plasma and periphery) PK model best fit lisinopril measurements, with two additional components, the gavage and urine. The absorption and renal clearance rates were similar between nonirradiated and irradiated animals (respectively: ratios 0.883, *p* = 0.527; 0.943, *p* = 0.605). Inter-compartmental clearance (from plasma to periphery) for the irradiated rats was lower than for the nonirradiated rats (ratio 0.615, *p* = 0.003), while the bioavailability of the drug was 33% higher (ratio = 1.326, *p* < 0.001).

**Interpretation:** Since receptors for lisinopril are present in endothelial cells lining blood vessels, and radiation induces vascular regression, it is possible that less lisinopril remains bound in irradiated rats, increasing circulating levels of the drug. However, this study cannot rule out changes in total amount of lisinopril absorbed or excreted long-term, after irradiation in rats.

## Introduction

There are no FDA-approved drugs to mitigate the delayed effects of radiation exposure that may occur after a radiological attack or nuclear accident (Singh et al., 2015b; Dicarlo et al., 2018). To date, angiotensin-converting enzyme inhibitors, a popular class of drugs commonly used to treat hypertension and heart disease (Riegger, 1989; Inagami 1999; Bicket 2002), are one of the most successful candidates for mitigation of radiation-induced injuries. They suppress the renin-angiotensin system (Bicket 2002) which regulates multiple physiological pathways (Inagami 1999; Rodgers and diZeraga, 2013). In preclinical models, radiation-induced injuries to the lung (Molteni et al., 2000; Kma et al., 2012; Medhora et al., 2012), kidney (Moulder et al., 2011; Fish et al., 2016), brain (Robbins et al., 2010) and hematopoietic tissues (Day et al., 2013; McCart et al., 2019, CM Orschell and GN Cox, personal communication) have been described to be mitigated by angiotensin converting enzyme inhibitors. There is also evidence that this class of drugs may mitigate delayed radiation injuries in humans treated with radiotherapy for cancer (Sun et al., 2018; Kharofa et al., 2012; Jenkins and Watts, 2011 and; Jenkins and Welsh, 2011).

Angiotensin-converting enzyme catalyzes the synthesis of a peptide, angiotensin II, which constricts blood vessels to increase blood pressure (Bicket 2002). Inhibition of the enzyme therefore blocks the constriction of blood vessels and lowers blood pressure. The enzyme is present on endothelial cells that line blood vessels (Heeneman et al., 2007). The lung, which is responsible for gas exchange between the air and the blood is rich in blood vessels and endothelial cells. Tissue distribution of lisinopril has been previously studied by planar anterior imaging in Sprague Dawley rats (Femia et al., 2008). A series of chelates were conjugated to lisinopril and their binding evaluated *in vitro* against purified rabbit lung angiotensin-converting enzyme. A lead conjugate was then labeled with technetium-99 m (^99m^Tc) and injected in rats to study uptake. In this study it was found that the drug bound significantly to the internal tissues, with over 18% of the signal recovered primarily in the lungs after 10 min, as compared to only 0.15% in the blood. Since radiation decreases vascular density in the lung and other organs, angiotensin converting enzyme and its activity is reduced in irradiated lungs (Ghosh et al., 2009). Similarly, well perfused organs such as the heart, gut, liver and kidney also have abundant endothelial cells which may decrease after irradiation (Baker et al., 2009; Stewart et al., 2010). It is not known how distribution of angiotensin-converting enzymes may be altered after radiation to these organs.

In order to test countermeasures for radiation-induced injuries to multiple organs after a radiological attack, total and partial body exposures are used in preclinical models (Singh et al., 2015a; MacVittie et al., 2019; Parker et al., 2019; Thrall et al., 2019). In rats, models of partial body irradiation sparing part of one hind leg (leg-out PBI) have been developed to simultaneously expose multiple organs to high doses of ionizing radiation without inducing hematological toxicity (Fish et al., 2016; Medhora et al., 2019). In this unique model, exposures between 9 and 14 Gy acutely damage the gut and bone marrow (acute radiation syndrome), followed by delayed injuries to the lung, heart, and kidney (Fish et al., 2016; Medhora et al., 2019). The acute radiation syndrome covers gastrointestinal injury between days 3–7, and hematopoietic cell depletion from days 8–30. Beyond day 30, rats experience delayed effects, with damage to the lungs, kidneys and other organs. Lung injury can be fatal at 13 Gy or higher to the thorax and typically occurs between days 40–90, while fatal renal injury manifests after >120-days (Fish et al., 2016).

To advance development of angiotensin-converting enzyme inhibitors as countermeasures for radiation damage, the FDA requires that their pharmacokinetics (PK) and pharmacodynamics (PD) be determined in irradiated subjects to understand how levels of the drug change with time after radiation (US FDA 2015). It is not known if such parameters are altered after leg-out PBI. Therefore, the current study evaluates PK of a lead angiotensin-converting enzyme inhibitor, lisinopril, as a step toward licensure for mitigation of radiation injury. The effect of radiation on PK of lisinopril was conducted at 35 days after radiation, since this time is within a latent window of injury in the model used. It does not coincide with lethal effects of the acute radiation syndrome or delayed effects of radiation that may only transiently interfere with oral drug delivery, absorption and metabolism. In addition, an angiotensin converting enzyme in the same family as lisinopril, enalapril, had efficacy to mitigate radiation pneumonitis when delivered as late as 35 days after radiation (Gao et al., 2013).

## Materials and Methods

### Animal Care

All animal protocols were approved by Institutional Animal Care and Use Committees (IACUC) at the Medical College of Wisconsin (MCW). Based upon direction from the IACUC, rats were designated as morbid and euthanized if they met specified veterinarian’s criteria as described previously (Medhora et al., 2015). WAG/RijCmcr rats bred at MCW were weaned to Teklad 8604 (Envigo, Madison WI) rodent diet along with hyper-chlorinated water. The rats were housed in a 14 h/10 h light/dark cycle, at 22°C with humidity maintained between 30 and 70%.

### Materials

Reagents were purchased from Sigma-Aldrich, St. Louis, MO. Solvents for liquid chromatography-mass spectrometry (LC-MS) analyses were of HPLC LC-MS grade. Enalaprilat (sc-205669) and (S)-Lisinopril-d5 (sc-220030) were purchased from Santacruz Biotechnology, Dallas, TX, United States.

### Experimental Procedures

Sample sizes are listed in Table 1.

**TABLE 1:** Sample sizes.

Group	Route	Administered lisinopril (mg/kg)	Plasma measurements	Urine measurements	Sample size
No radiation	Gavage	0	1	0	4
No radiation	Gavage	300	0	1	4
No radiation	Gavage	300	1	0	55
No radiation	Gavage	300	2	0	2
No radiation	Gavage	600	0	1	6
No radiation	Gavage	600	1	1	3
No radiation	Gavage	600	2	0	9
No radiation	Gavage	600	4	0	9
No radiation	IV	60	0	1	6
No radiation	IV	60	1	0	11
No radiation	IV	60	1	1	3
No radiation	IV	60	2	0	3
Radiation	Gavage	300	0	1	5
Radiation	Gavage	300	1	0	58
Radiation	Gavage	300	2	0	2
Radiation	Gavage	600	0	1	6
Radiation	Gavage	600	2	0	8
Radiation	Gavage	600	4	0	8
Radiation	IV	60	0	1	6
Radiation	IV	60	1	0	11

#### Leg–Out PBI in Rats

WAG/RijCmcr female rats were irradiated without the use of anesthetics at 11–12 weeks of age weighing ∼155 g. All irradiations were done between 8–11 am. For leg-out PBI, non-anesthetized rats were immobilized in a plastic jig and irradiated using a XRAD-320 orthovoltage x-ray system (Precision X-Ray, North Branford, Connecticut). The x-ray system was operated at 320 kVp and 13 mA, with a half value layer of 1.4 mm Cu and a dose-rate of 1.75 Gy min^−1^ for total doses of 12.5 or 13 Gy. During the irradiation, each rat was confined in a chamber which allows irradiation of two rats simultaneously. One hind limb of each rat was carefully externalized from the chamber and shielded with a 0.25-inch lead block. The dose to this leg was 2 Gy. The dual-chambered jig was placed on a plane perpendicular to the beam direction, with distance from source to the midline of rats set at 61 cm. Collimator jaws and dosimetry were used as previously described (Medhora et al., 2014). The irradiation field at midline was large enough to cover both chambers with adequate (at least 2 cm) margins. Supportive care was provided to rats receiving 13 Gy. Supportive care consisted of delivery of the antibiotic Enrofloxacin (10 mg/kg/day) from days 1–14 in the drinking water, and hydration by daily subcutaneous injection of saline (40 ml/kg/day) from days 3–7, post-irradiation.

#### Administration of Lisinopril: Gavage and Intravenous Injection

At 35 days post-irradiation, irradiated rats or age-matched controls were administered a single dose of lisinopril (21CEC PX Pharm Ltd. United Kingdom; dissolved in filtered reverse-osmosis water). Depending on treatment group, lisinopril was administered either by oral gavage or an intravenous (IV) injection via the tail vein. For oral gavage, the rats were manually restrained, and a gavage needle attached to a syringe was inserted into the esophagus and 0.4 ml of either 0.3 mg/rat or 0.6 mg/rat lisinopril was delivered. A different group of rats received lisinopril at a diluted (1:10) dose of 0.06 mg/rat injected IV via the tail vein. The rats were manually restrained, and facilitation of tail vein dilation was achieved with dipping the tails in warm water. Once the veins were dilated, a 25-gauge needle attached to a syringe was inserted into the tail vein and 0.4 ml of lisinopril administered.

#### Blood Collection

Blood was collected via the jugular vein in order to measure lisinopril levels in the plasma at various timepoints. Rats were anesthetized with 3–5% isoflurane, the forelimbs restrained in the caudo-dorsal direction and a 23-gauge needle inserted into the center of the jugular fossa by a trained technician. The needle and syringe were coated with EDTA and ∼0.5 ml blood was collected at each timepoint. Platelet free plasma was obtained by first centrifuging the blood at 1,000 x g for 15 min and re-centrifuging the supernatant at 10,000 x g for 10 min. All centrifugations were carried out at 4°C.

#### Urine Collection

Urine was collected in a Nalgene rat metabolic cage. A rat was placed in the metabolic cage with access to food and water for 24 h. After 24 h urine volume was recorded, and an aliquot was frozen and stored at −80°C for LC-MS analyses.

### Measurements of Lisinopril

At day 35 after irradiation, all rats received a single gavage or intravenous dose of lisinopril (0.3 or 0.06 mg). Plasma was measured 1–4 times in each animal, at 0, 0.5, 1, 1.5, 2, 2.5, 3, 4, 5, 6, 8, 24 or 48 h after oral gavage, and 5 min, 1.5 and 24 h after intravenous injection. The renal clearance (amount excreted in urine) was measured 24 h after either gavage or injection. Terminal measurements of lisinopril in the kidney, liver and lung were made at 5 min and 1.5 h after IV injection of lisinopril. Measurements of lisinopril in the lungs and kidneys were performed after 24- and 48-h following gavage administration.

#### Determination of Lisinopril Levels in Rat Plasma or Urine by LC-MS/MS

Aliquots (0.1 ml) of rat plasma or urine were extracted with ∼3 volumes of cold acidified methanol spiked with enalaprilat as an internal standard (0.3 ml of methanol, 20 µL of 0.1 M HCl and 3 µL of 0.1 mM enalaprilat), mixed well and allowed to stand for 5 min before centrifugation at 14,000 rpm for 5 min at 4°C. The supernatant was passed through a Phree phospholipid removal plate (Phenomenex) and the eluate dried completely under a flux of air and reconstituted with 120 µL of LC-MS mobile phase (5% acetonitrile, 95% water, 0.1% formic acid), spiked with 1 µM of lisinopril-d_5_ used as a second internal standard. The sample was vortexed thoroughly for 15 min at 4°C and centrifuged for 30 min at 20,000 g. 80 µL of the supernatant was transferred to HPLC autosampler vials and processed for lisinopril analyses by LC-MS/MS. The analyses were performed using a Shimadzu Nexera-2 UHPLC system coupled to Shimadzu LCMS-8030 triple quadrupole mass detector. Samples were injected into C_18_ reversed phase column (Waters Cortecs UPLC C_18_ 2.1 mm × 50 mm, 1.6 µm) thermostated at 40°C and equilibrated with 0.1% formic acid in water:acetonitrile (95:5). Compounds were eluted by increasing the concentration of acetonitrile in the mobile phase from 5 to 40% over 2.5 min at the flow rate of 0.5 ml/min. Detection was carried out using electrospray ionization (ESI) source in the multiple reaction monitoring (MRM) mode, using the following transitions: 406.1 > 84.1 (lisinopril), 411.1 > 84.1 (lisinopril-d_5_), and 349.0 > 206.1 (enalaprilat).

#### Determination of Lisinopril Levels in Rat Lung, Liver, and Kidney Tissues

The lungs, liver and kidneys were harvested, weighed, and powdered in liquid nitrogen. A total of one lung (left), one lobe of liver (middle) and one kidney (right)/rat was used for extraction. To the pulverized tissue, 1 ml of cold DPBS was added, vortexed well and extracted with 3.23 ml of acidified methanol containing enalaprilat as internal standard (3 ml of methanol, 200 µL of 0.1 M HCl, 10 µL of 0.1 mM enalaprilat, and 20 µL of water). The sample was incubated overnight on a shaker at 4°C. The extract was then centrifuged at 14,000 rpm for 5 min at 4°C. The supernatant was passed through a Phree phospholipid removal plate and the eluate dried under a flux of air. The dried residue was reconstituted and analyzed by LC-MS/MS as described above for plasma/urine samples.

#### Determination of Efficiency of Extraction of Lisinopril

The efficiency of extraction of lisinopril from blood, plasma, urine, lungs, liver and kidney samples was estimated using spike-in experiments. Age-matched naïve rats (*n* = 3–5) that were not irradiated, were used for this study. A known volume of lisinopril from a stock of 1 mg/ml was added to a known volume of harvested blood, plasma or urine *in vitro*. Similarly, a known volume of stock lisinopril was added to a measured aliquot of suspension containing pulverized lung, liver or kidney in DPBS as described above. The samples were then analyzed by LC-MS/MS as already described for plasma and tissues, after again adding enalaprilat as an internal standard. The estimated amount of lisinopril in each sample was compared with the actual amount used to spike the same sample. The ratios were used to determine the efficiency of extraction. For modeling, the measured lisinopril concentrations/amounts were divided by the corresponding extraction efficiencies.

### Measurement of Kidney Function

Previous published work has shown that rising blood urea nitrogen (BUN) levels are superior to histopathology for assessing kidney injury (Moulder et al., 2011) To measure BUN, rats were anesthetized with 3–5% isoflurane for blood draws conducted by an experienced technician. The BUN was assayed from serum as described previously (Cohen et al., 1994; Medhora et al., 2014) using a urease-nitroprusside colorimetric assay. BUN values were expressed as mg/dL of serum and means with 95% confidence intervals were used for statistical analysis. Urine protein (UP) and creatinine (UC) were also measured as described (Moulder et al., 2011). The UP/UC ratio is used as a sensitive indicator of kidney function to measure urine-concentrating defects that occur upon renal radiation injury and to normalize for animal size differences.

### Statistical Methods

#### Non-compartmental Estimates

Non-compartmental estimates based on gavage-administered plasma concentrations were computed using the R package PK version 1.3.5. The concentrations were normalized to 300 µg of drug administered, and time was measured in hours. The AUC 0-tlast was calculated using the linear trapezoidal rule on the arithmetic means at the different time points. Bootstrap t confidence intervals are reported.

#### Two-Compartment Pharmacokinetics Model

**FIGURE 1:**
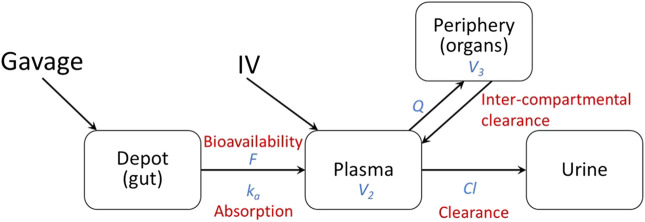
Diagram of two-compartment fitted model.

Pharmacokinetic (PK) modeling was performed on the data to measure distribution of the drug in different tissues (compartments). The two compartments fitted with observed data were the central compartment (plasma) and the peripheral compartment (internal tissues such as lungs, liver, kidneys, etc.). Administration by gavage was modeled using the gut as a depot to include bioavailability (*F*) and absorption (*k*_*a*_) from the gut (Figure 1). The diffusion of lisinopril between the plasma and peripheral compartments was modeled by Q, the inter-compartmental clearance rate, where larger numbers reflect more diffusion between the two compartments. Clearance out of the system was modeled by Cl, the renal clearance rate via urine (Figure 1). The model was fitted using the open-source R package Nonlinear Mixed-Effects Model Development and Simulation (nlmixr) along with related R packages.

More specifically, the following differential equation system was fitted:

$$\begin{array}{l} \frac{\text{d}}{\text{d}t}\left(Y_{\text{depot}}\right)=-k_{a}*Y_{\text{depot}},\\ \frac{\text{d}}{\text{d}t}\left(Y_{\text{plasma}}\right)=+F*k_{a}*Y_{\text{depot}}-Cl*C_{\text{plasma}}-Q*C_{\text{plasma}}+Q*\frac{Y_{\text{peri}}}{V_{3}},\\ \frac{\text{d}}{\text{d}t}\left(Y_{\text{peri}}\right)=+Q*C_{\text{plasma}}-Q*\frac{Y_{\text{peri}}}{V_{3}},\\ \frac{\text{d}}{\text{d}t}\left(Y_{\text{urine}}\right)=+Cl*C_{\text{plasma}}. \end{array}$$

where $Y_{\text{depot}}$, $Y_{\text{plasma}}$, $Y_{\text{peri}}$, and $Y_{\text{urine}}$ are the amount of lisinopril in the gut (depot), plasma (central), peripheral, and urine compartments, respectively, in µmoles; V_2_ and V_3_ are the apparent volumes of the plasma and the peripheral compartments in liters (L); and $C_{\text{plasma}}=Y_{\text{plasma}}/V_{2}$ is the concentration of lisinopril within the plasma compartment in µmoles/L.

An additive error with compartment-specific variance was assumed for the plasma, peripheral, and urine compartments. Multiple measurements from the same animal were linked via a log-normally distributed multiplicative random effect on the compartment volumes *V*_2_ and *V*_3_.

The observed measurements are the concentration in plasma, $C_{\text{plasma}}$ and the (cumulative) amount in the urine, $Y_{\text{urine}}$. The administered lisinopril amount (in g) was converted to moles via dividing by its mass (405.5 g/mol).

The model was fitted to the plasma and urine data, the accumulation in the peripheral compartment was inferred from the model.

## Results

Non-compartmental (AUC) estimates showed significantly higher lisinopril circulating in irradiated animals over the first 24 h (radiation/no radiation ratio 1.42, *p* < 0.0001). When the same model was plotted on a log scale, the lisinopril in both irradiated and non-irradiated animals did not reach zero, indicating the existence of at least one other internal compartment in the PK model.

**FIGURE 2:**
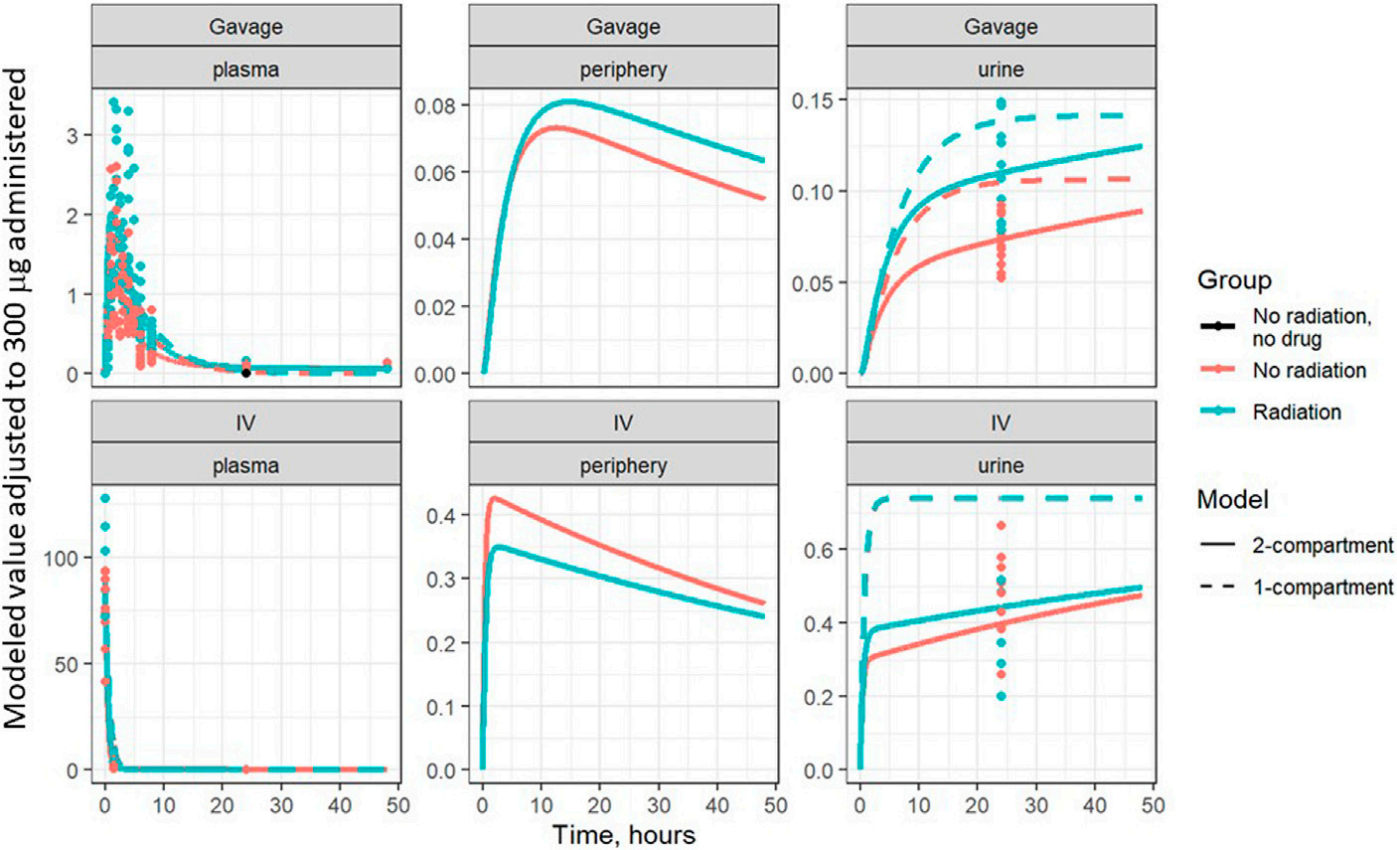
Lisinopril over time in plasma, periphery, and urine, broken out by delivery technique, plotted on a linear scale. Points are measured data values. Solid curves are fitted values from the two-compartment model, not adjusted for bioavailability. Dashed lines represent the best-fit one-compartment model.

**FIGURE 3:**
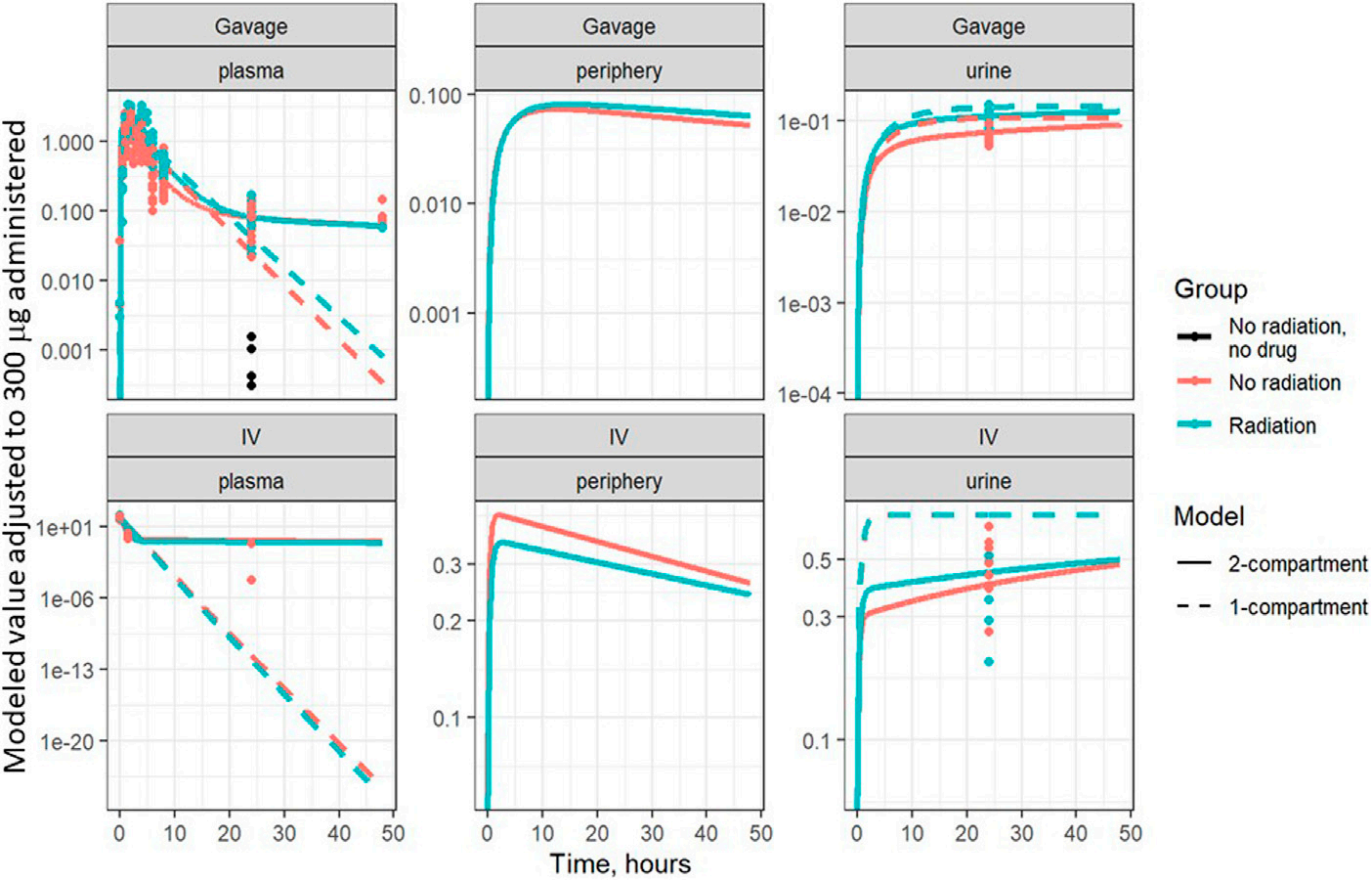
Same data as Figure 2, plotted on a log scale. Points are measured data values. Solid curves are fitted values from the two-compartment model, not adjusted for bioavailability. Dashed lines represent the best-fit one-compartment model.

Based on visual predictive checks and a formal likelihood ratio test (*p* < 0.001), a two internal-compartments PK model including plasma and peripheral compartments, with two additional external compartments to model gavage and urine, best fit the plasma and urine concentrations (see Figures 2, 3). The goodness-of-fit was quantified as *R* ^2^ = 90.1%. The dashed lines in Figures 2, 3 show the best-fit one-compartment model. Compared to this, the two-compartment model shown by solid lines better fit the data measured in the urine (Figure 2) and plasma (Figure 3) especially at later time points.

The model returned two kinds of parameters, the base rates in non-irradiated animals (Table 2) and the ratio of rates for irradiated animals (Table 3). The model parameters for irradiated animals can be calculated using the base rate estimate from Table 2 multiplied by the corresponding ratio from Table 3.

**TABLE 2:** Two-compartment model base parameter estimates for control group, including 95% confidence interval bounds; fitted to plasma + urine data. L represents liters in the units column.

Parameter	Estimate	Lower 95% Cl	Upper 95% Cl	Units
Absorption rate	0.279	0.197	0.395	1/hr
Renal clearance rate	0.009	0.008	0.011	L/hr
Central volume	0.008	0.007	0.010	L
Inter-compartmental clearance rate	0.014	0.011	0.017	L/hr
Peripheral volume (lung)	0.513	0.360	0.730	L
Bioavailability	0.192	0.167	0.221	Scalar

**TABLE 3:** Two-compartment model parameter ratio estimates for radiation group, including 95% confidence interval bounds and *p*-values; fitted to plasma + urine data.

Parameter	Estimate	Lower 95% Cl	Upper 95% Cl	*p*-value
Absorption rate ratio	0.883	0.599	1.300	0.527
Renal clearance rate ratio	0.943	0.756	1.177	0.605
Inter-compartmental clearance rate ratio	0.615	0.445	0.850	0.003
Bioavailability ratio	1.326	1.142	1.538	<0.001

This model yielded estimates for the ratios of absorption, clearance, inter-compartment clearance, and bioavailability (proportion of the dose that reaches the systemic circulation) of lisinopril between non-irradiated and irradiated rats. *p*-values were calculated based on whether the ratio differed significantly from 1. The absorption and renal clearance rates were similar between non-irradiated and irradiated animals (respectively: ratio 0.883, *p* = 0.527; ratio 0.943, *p* = 0.605). The inter-compartmental clearance for the irradiated rats was significantly lower than for the non-irradiated rats (ratio 0.615, *p* = 0.003), while the bioavailability of the drug was 33% higher (ratio = 1.326, *p* < 0.001) (see Table 3).

**FIGURE 4:**
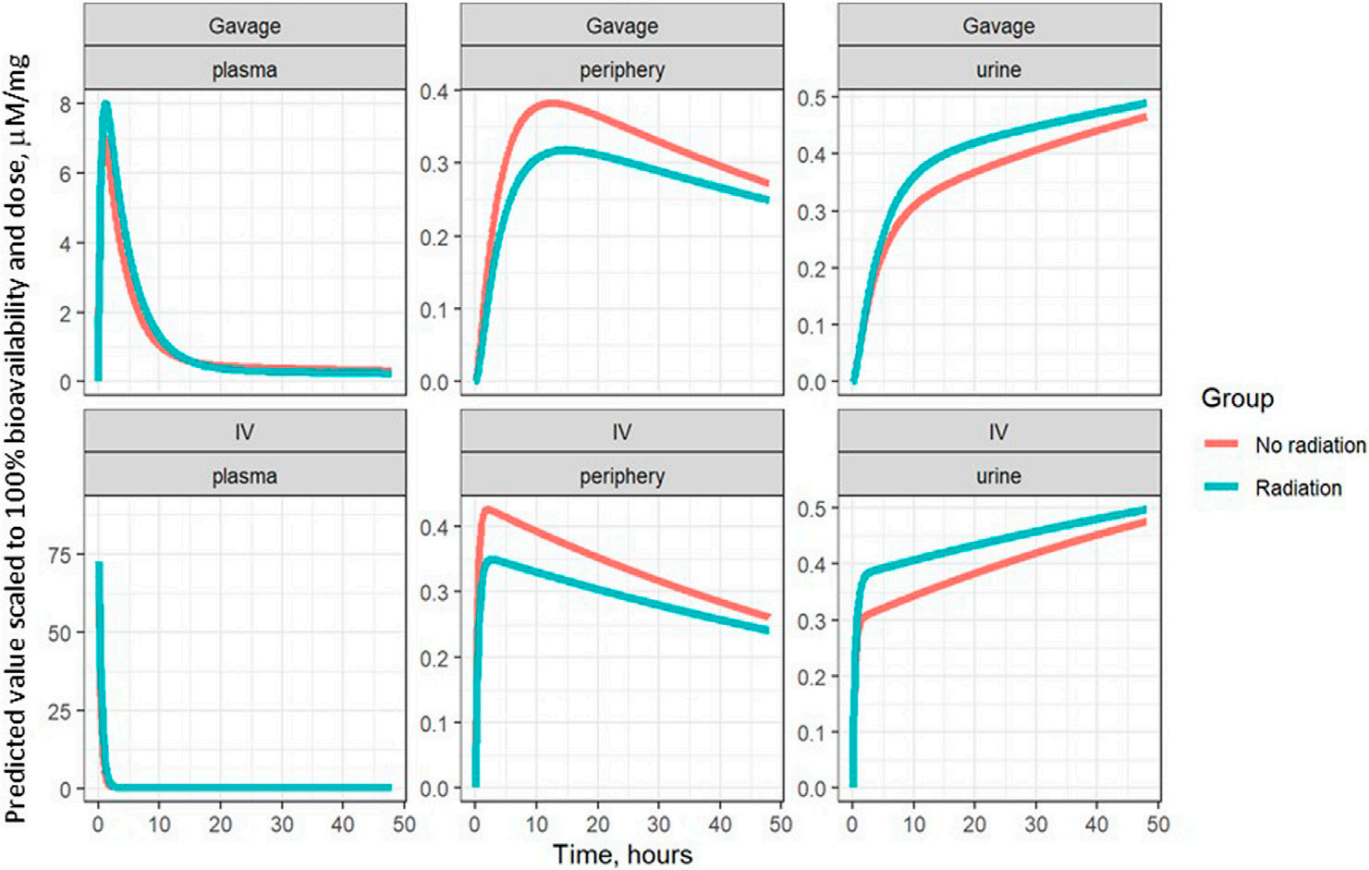
Model-inferred estimates for lisinopril over time in plasma, lungs, and urine, adjusted for bioavailability, broken out by delivery technique.

The model parameters were used to plot inferred amounts of lisinopril in plasma, the peripheral compartment, and urine over time. Figure 2 shows the model-inferred curves fitted to the measured data values. Although the urine sample was collected only once in a subset of animals, this data was crucial for the ability of the model to separate bioavailability from absorption rate (the urine measurements broken out by delivery technique and group are shown in Table 4). Note that the models in Figure 2 have not been adjusted for bioavailability, leading to the appearance that the lisinopril retained in irradiated tissue differs by delivery technique. Figure 4 shows the model-inferred curves adjusted for equivalent bioavailability, by dividing the model output by the relevant bioavailability estimate (0.192 for non-irradiated rats and 0.254 for irradiated rats) (see Tables 2, 3). This demonstrates that the delivery technique does not affect the amount of lisinopril retained in irradiated tissue.

**TABLE 4:** Measured lisinopril excreted in urine per 300 µg administered (µmoles).

Route	Group	Sample size	Geometric mean (µmoles)	Standard deviation (µmoles)
Gavage	No radiation	13	0.072	0.013
Gavage	Radiation	11	0.11	0.026
IV	No radiation	9	0.46	0.13
IV	Radiation	6	0.37	0.14

**FIGURE 5:**
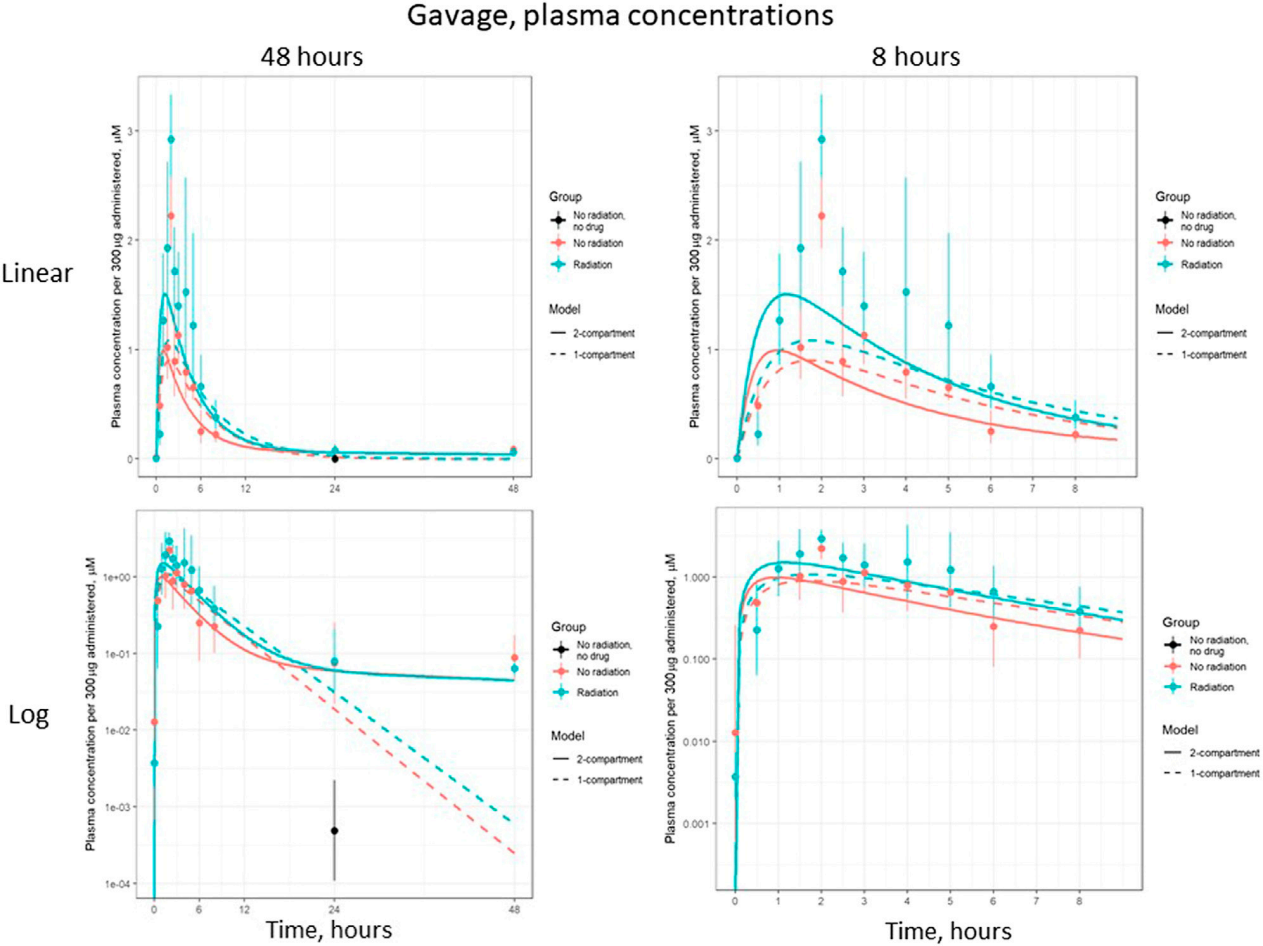
Plasma concentration of lisinopril (geometric mean ± SD) when administered by gavage, broken out by time and scale.

Figure 5 shows the plasma concentration of gavage-administered lisinopril over 48 and 8 h, on both log and linear scales. The difference in peak concentration shows the higher bioavailability of lisinopril in irradiated vs non-irradiated rats. The log-scale plots make clear that the plasma concentration never reaches zero.

**FIGURE 6:**
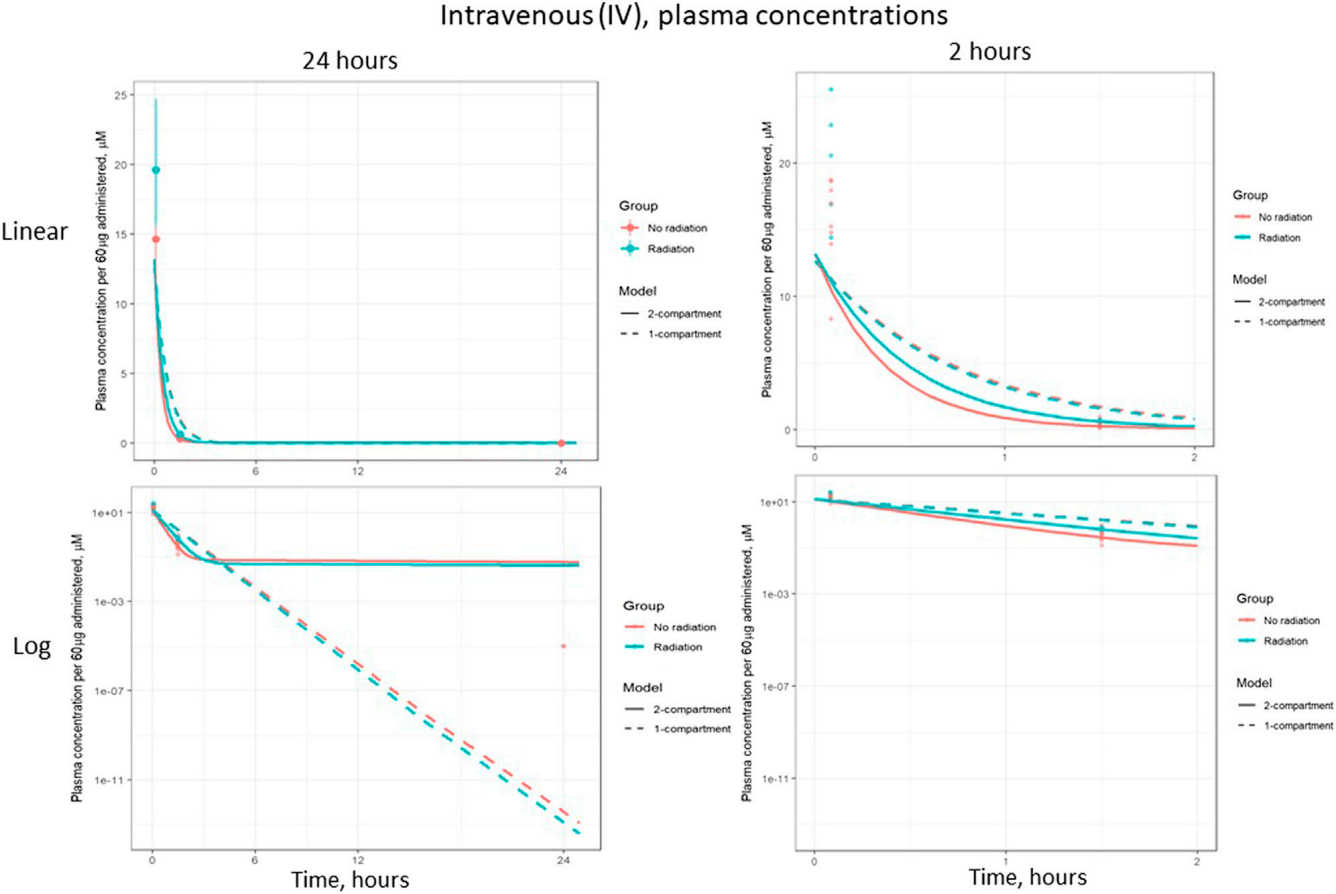
Plasma concentration of lisinopril (geometric mean ± SD) when administered by intravenous (IV) injection, broken out by time and scale.

Figure 6 shows the plasma concentration of IV-administered lisinopril over 24 and 2 h, on both log and linear scales. The difference in plasma concentrations seen after 5 h reflects the reduced inter-compartmental clearance rate, i.e., there is less lisinopril in the plasma because less is being cleared to the plasma from the peripheral compartment. This difference is less apparent in the gavage data due to the increased bioavailability of gavage-administered lisinopril in irradiated rats.

**FIGURE 7:**
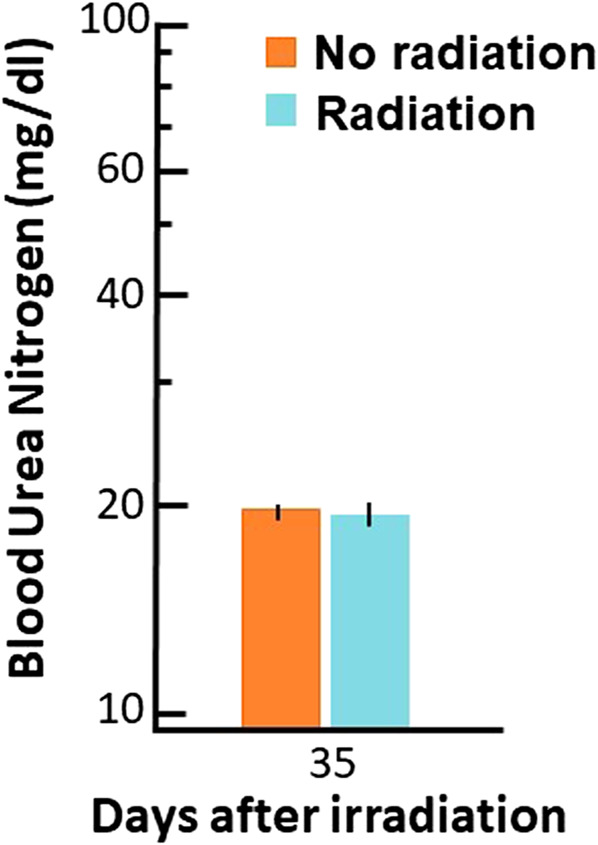
Blood urea nitrogen (BUN, mg/dl (log scale on *Y*-axis)) values at 35 days after radiation. Data are shown as means and 95% confidence intervals.

Finally, BUN values (see Materials and Methods) were used to infer renal function at the same timepoint at which the PK studies were conducted. The results are plotted in Figure 7. There was no difference in BUN between irradiated and non-irradiated rats indicating renal function was not changed at 35 days after irradiation. Another sensitive measure of renal function, the urine protein to urine creatinine ratio (UP/UC), did not differ between irradiated rats at 35 days after irradiation (0.20 (0.22–0.31)) compared to non-irradiated control rats (0.24 (0.19–0.28)).

## Discussion

The non-significance of the difference in absorption rates paired with the significant increase in bioavailability suggests that radiation increases the bioavailability of lisinopril independently of its absorption from the gut. Further studies specifically designed to measure absorption are needed to confirm this result, since irradiation is known to breach the integrity of the intestinal barrier (Booth et al., 2012). However, gastrointestinal injury peaks within 7 days after irradiation in the rat (Fish et al., 2016; Fish et al., 2020), so it is possible that the injury is repaired (at least partially) by 35 days when the current study was conducted.

The reduction in inter-compartmental clearance suggests that circulating lisinopril is cleared more slowly from the central plasma compartment in irradiated animals. However, from the results presented here we cannot determine if radiation interferes with lisinopril leakage/diffusion into the periphery or reduced lisinopril is bound to the vasculature, especially in the peripheral compartments such as the lung, liver and kidney, which are known to be well perfused with blood. Since lisinopril has been shown to bind substantially to the peripheral compartment in the absence of radiation (Femia et al., 2008), the latter explanation is consistent with the binding of lisinopril to angiotensin converting enzyme (its receptor) found on vascular endothelial cells lining the blood vessels. Since radiation induces vascular regression in organs and tissues (Baker et al., 2009; Ghosh et al., 2009; Stewart et al., 2010), irradiated rats may have fewer receptors leaving more unbound lisinopril to circulate in the blood.

Lisinopril is not known to be metabolized *in vivo*, but instead removed primarily by excretion via the kidney (Beermann 1988). Since the renal clearance rates were similar between nonirradiated and irradiated animals we checked the kidney function in these rats to confirm renal function had not changed. Levels of BUN and UP/UC are commonly used as a biomarker to follow renal function and are actually superior to histopathology for assessing radiation nephropathy in irradiated WAG/Rij rats (Moulder et al., 2011). As with renal clearance, the BUN and UP/UC were not different at 35 days after irradiation (Figure 7). It should be noted that BUN levels and UP/UC ratio do ultimately rise after radiation (by 90 days) in the same rat model (Fish et al., 2016).

The PK of lisinopril has been described in humans and are somewhat comparable to the results described in this paper. The peak serum levels in humans are 6–8 h (Beermann 1988), compared to 2–3 h in rats in the current study. Though bioavailability was increased by radiation, the model-based circulating half-life of the drug remained similar in irradiated (2.5 h) and non-irradiated (2.8 h) rats. The inter-individual variation was 4-6-fold in humans (Beermann 1988) and 3-4-fold in nonirradiated rats injected with lisinopril (result not shown). Variation in irradiated rats at 90 min was 1.8-fold after IV injection. After oral administration by gavage, variation was 1.6-fold and 2.7-fold in non-irradiated and irradiated rats respectively at 90 min (data not shown). Similar to the multiple phasic plots observed in Figures 2–6, a polyphasic decrease in circulating lisinopril over time occurred in humans. Both species demonstrated an initial linear drop followed by a slower terminal phase (Beermann 1988, Figure 5). The prolonged terminal phase in humans (half-life of 46.7 h) was postulated to be due to binding of lisinopril to angiotensin-converting enzyme (Beermann 1988). The model-based estimate of the terminal half-life is 65 h in nonirradiated rats and 83 h in irradiated rats in this study, and also could be postulated to be due to the tight binding of lisinopril to its receptor, angiotensin converting enzyme.

In summary, irradiation of multiple organs increases circulating levels of lisinopril when administered at 35 days after exposure. Statistical modeling suggests that this is caused by a decreased amount of lisinopril distributed in the periphery of irradiated rats. Since lisinopril is known to bind with high affinity to angiotensin converting enzyme, which is present on cells lining the blood vessels, the vascular compartment of the periphery is the most likely site to hold this bound lisinopril. Though the current study suggests the rates of absorption and clearance of lisinopril are not altered at 35 days after radiation, further studies specifically targeting such measurements must be conducted for confirmation. Absorption over a longer time (but at the same rate) in irradiated rats given gavage, could result in increased bioavailability, and cannot be ruled out since excretion from the gut into the feces was not measured in this study. Also, the model-based terminal renal excretion was prolonged (83 vs. 65 h) in irradiated rats, indicating that clearance may be altered. In addition, since the gut is injured between 6 and 10 days (Booth et al., 2012; Fish et al., 2016; Fish et al., 2020) and the kidney after 90 days (Fish et al., 2016; Fish et al., 2020) post-irradiation, it is also possible that the PK of lisinopril will be different around these time points.

Lisinopril is widely used to regulate blood pressure or treat cardiovascular disease. Angiotensin-converting enzyme expression is increased in cardiac fibrosis and disease (Harada et al., 1999; Dilsizian et al., 2007). The results from radiation injury in the current study indicate the possibility that bioavailability may be altered by other pathological conditions as well. The unique result of increased bioavailability in this study after radiation is consistent with a reduction in blood vessel density, which has been previously described (Baker et al., 2009; Ghosh et al., 2009; Stewart et al., 2010).

## Data Availability

The raw data supporting the conclusions of this article will be made available by the authors, without undue reservation.
